# Effects of *Fagonia indica* on Letrozole-Induced Polycystic Ovarian Syndrome (PCOS) in Young Adult Female Rats

**DOI:** 10.1155/2022/1397060

**Published:** 2022-05-26

**Authors:** Anam Younas, Liaqat Hussain, Arham Shabbir, Muhammad Asif, Musaddique Hussain, Faiza Manzoor

**Affiliations:** ^1^Department of Pharmacology, Faculty of Pharmaceutical Sciences, Government College University Faisalabad, Faisalabad, Pakistan; ^2^Institute of Pharmacy, Faculty of Pharmaceutical and Allied Health Sciences, Lahore College for Women University, Lahore, Pakistan; ^3^Department of Pharmacology, Faculty of Pharmacy, Islamia University Bahawalpur, Bahawalpur, Pakistan; ^4^Department of Nutritional Sciences, Government College University Faisalabad, Faisalabad, Pakistan

## Abstract

Polycystic ovarian syndrome is a multidisciplinary endocrinopathy of reproductive-aged women that provokes insulin resistance, hyperandrogenism, cardiovascular problems, obesity, and menstrual complications. The present study was designed to investigate the effectiveness of ethanolic extract of *Fagonia indica* in letrozole-induced PCOS young adult female rats. HPLC was carried out to find the phenolic and flavonoid content of the ethanolic extract of *Fagonia indica*. Twenty-five female rats were taken and initially divided into two groups: group I (control group) and group II (PCOS group). PCOS was induced by letrozole given orally by gavage. Body weight was recorded weekly and vaginal cytology was analyzed daily. After induction of disease, the PCOS group is further divided into four groups (*n* = 5): group II (positive control with PCOS), group III (metformin 20 mg/kg treated group), group IV (ethanolic extract of *Fagonia indica* 500 mg/kg treated group), and group V (metformin plus *Fagonia* extract). At the end of experimental period, the blood sample of each rat was collected and serum was separated by centrifugation. Afterwards hormonal analysis, lipid profile and liver functioning tests were performed. Ovaries were removed and preserved for histopathological findings while the liver of each rat was stored for the determination of antioxidant potential assessment. *Fagonia indica* was found to possess quercetin as one of the major flavonoid phytoconstituents. The plant extract exhibited its beneficial effects by restoring hormonal balance, lipid profile, and liver functioning markers. Treatment with *F*. *indica* reduced body weight, resolved ovarian cysts, and showed positive effects on follicular growth. Treatment with plant also increased the levels of antioxidant enzymes. This study validates the potential of *Fagonia indica* for the amelioration of metabolic, as well as, hormonal disturbances that occurred in PCOS.

## 1. Introduction

Hyperandrogenemia is a salient feature of PCOS, and a major contributor to cosmetic anomalies including hirsutism, acne, and male pattern alopecia in affected women. Polycystic ovarian syndrome is a complex endocrine and metabolic disorder [1] that is characterized by multiple follicular cysts, found on the periphery of ovaries and can be visualized ultrasonographically. Overweight and obesity further complicate the situation by accelerating insulin resistance [2]. Moreover, PCOS is associated with different endocrine, metabolic, and reproductive traits including hyperandrogenism, hyperinsulinism, obesity, hyperlipidemia, type 2 diabetes mellitus, cardiovascular abnormalities, depression, anxiety, anovulation, oligo-ovulation, infertility, hirsutism, and acne [3, 4]. This syndrome is so devastated that it can cause endometrial hyperplasia and subsequently the development of endometrial cancer [5]. Concurrently, the PCOS patients are also suffering from elevated levels of low-density lipoprotein (LDL), triglycerides, total cholesterol, and decreased level of high-density lipoprotein (HDL) [6]. In PCOS women, the balance of sex hormones, e.g., luteinizing hormone (LH), follicle-stimulating hormone (FSH), estrogen, progesterone, and testosterone is disturbed [7]. Blood glucose levels are also elevated because of insulin insensitivity. Evidence suggested that increase in GnRH pulse frequency and amplitude from hypothalamus stimulates LH synthesis over FSH, which results in elevated LH/FSH ratio in PCOS women. Consequently, enhanced luteinizing hormone levels begin to develop metabolic and reproductive disorders such as increased androgen synthesis in theca cells of ovaries, impairment of FSH and estrogen synthesis, and promoting the secretion of insulin-like growth factor-1 (IGF-1). Nowadays, polycystic ovarian syndrome (PCOS) has become very common among females of reproductive ages. It is a multidisciplinary disorder and hence requires a multidisciplinary consultation to manage the symptoms of the disorder [1].

Lifestyle modifications for PCOS treatment in obese patients include moderate exercise (≥30 min/day), reduce psychological stressors, weight reduction, calorie deficit diet of 500–1000 kcal/day, decrease caffeine consumption, and low glycemic index (GI) diet [8]. Current treatment modalities include metformin or OCP (estrogen-progestin combinations), antiandrogens, flutamide, pioglitazone, spironolactone, cyclic-progestins, GnRH agonists, myoinositol, and fertility treatments such as clomiphene citrate and letrozole [2, 9–11]. When used for longer duration, each of the above treatment has adverse effects that limit their use in the patients. For example, metformin causes fatal and nonfatal lactic acidosis in 1–17/100,000 patients per year. Oral contraceptive pills (OCPs) are associated with weight gain, thromboembolism, and cardiovascular events. Some side effects are more common in patients using OCPs such as abdominal pain, dysmenorrhea, nausea, headache, backache, and dizziness [12]. Antiandrogenic agents are hepatotoxic that could be very fatal [13].

Nowadays, many physicians and patients are inclined towards herbal treatment due to these abovementioned adverse effects of conventional treatment and cost of the treatment. The pharmacological and therapeutic effectiveness of medicinal plants is due to the presence of different phytoconstituents in these plants and these can be used for various ailments [14, 15]. In this respect, herbal remedies for PCOS are very effective and some of these herbal therapies reduce hyperandrogenism and improve ovulation and insulin sensitivity without causing the abovementioned adverse effects. This is why people tend to use these herbal remedies to treat PCOS. Some of these herbal interventions include *Glycyrrhiza glabra*, *Mentha spicata*, *Panax ginseng*, flaxseed, *Aloe vera*, *Chaste berry*, *White poeny*, cinnamon, milk thistle, *Matricaria chamomilla*, N-acetyle cysteine, D-chiro-inositol, Kacip fatimah, *Astragalus* polysaccharide, *Apium graveolens*, and *Cinnamon zeylanicum* [16, 17].

*Fagonia indica* is a member of Zygophyllaceae family. All of the species of this family are herbs and shrubs/shrublets [18]. *Fagonia* is commonly known as dhamasa booti. *Fagonia* has a great medicinal potential to treat a lot of diseases. It is known to possess following activities such as antioxidant, thrombolytic, antimicrobial, antidiabetic, antitumor, anti-inflammatory, hepatoprotective, antihaemorrhagic, laxative, and antiulcerogenic potential. *Fagonia* has been used traditionally for the treatment of menstrual problems for thousands of years. Practitioners of traditional medicine have used this plant for the treatment of PCOS in different areas of Pakistan. Due to its folklore use in PCOS, it is worthwhile to explore its potential in PCOS treatment. The current study investigates the efficacy of ethanolic extract of *Fagonia indica* for the treatment of letrozole-induced PCOS rats.

## 2. Material and Methods

### 2.1. Plant Collection and Authentication

*Fagonia indica* was collected from a town called Tarnol Phatak situated in Islamabad, Pakistan. The plant was collected in March 2021. It was identified and authenticated by the botanist, Dr. Mansoor Hameed at University of Agriculture Faisalabad and was preserved under a specific specimen number 071-21-01 in the herbarium of Department of Botany, University of Agriculture Faisalabad, for the further reference.

### 2.2. Preparation of Plant Extract

The extraction was carried out by the simple maceration method. The plant was air dried and soaked for 14 days in absolute ethanol [19] in 1 : 5 ratio. Subsequently, it was filtered by using a Whatman filter paper and the resultant filtrate was evaporated by using a rotatory evaporator (SCILOGEX RE100-pro 5 L). At the end of this process, a semisolid extract was obtained that was administered to PCOS-induced rats for experimental purpose.

### 2.3. Plant Characterization

HPLC was carried out to find the phenolic and flavonoid content of the ethanolic extract of *Fagonia indica*.

### 2.4. Total Phenolic Content (TPC)

Total phenolic content was measured by using Folin–Ciocalteu reagent. 0.5 mL solution of the extract (0.05 g/5 mL) was admixed with 0.5 mL of Folin–Ciocalteu reagent and then added 7.5 mL of deionized water. This mixture was allowed to set at 25°C for 10 min after that 1.5 mL of 20% sodium carbonate (w/v) was added. Then, the mixture was heated in a water bath at 40°C for 20 min and cooled it in ice bath. Finally, the absorbance was recorded at 755 nm wavelength by using a spectrophotometer. Gallic acid was used as a standard, so the total phenolic contents were calculated by using the standard curve of gallic acid (100–1300 ppm). TPC was measured as gallic acid equivalent (GAE), and the results were expressed as mg/gm of dry matter [20].

### 2.5. Total Flavonoid Content (TFC)

The method described by Dewanto [21] was adopted to determine total flavonoid contents. The mixture was kept at room temperature for 5 min and 0.6 mL of 10% A1C1_3_ was added. The mixture was again kept for 5 min at room temperature. After 5 min, 2 mL of 1 M NaOH was mixed and distilled water was used to make up the volume. The absorbance was recorded at 510 nm by using a spectrophotometer. Catechin was used as a standard, so the total flavonoid contents were calculated by a calibration curve for catechin (10–130 ppm). The TFC was measured as catechin equivalent (CE) [22].

### 2.6. Housing of Animals

Twenty-five healthy female Wistar albino rats (150–200 grams) were acquired and kept in the animal house of the Faculty of Pharmaceutical Sciences, Government College University of Faisalabad (GCUF). Standard conditions (12 hrs light/dark cycles at room temperature 25 ± 5°C with 45–55% humidity) were maintained. Standard laboratory diet and water ad libitum were given to rats. The experiments on animals were performed according to the guidelines of the Ethical Review Committee ERC-21-886, Government College University, Faisalabad. The experimental design was approved by Advanced Studies and Research Board (ASRB) under the study number IRB-21-19886.

### 2.7. Induction of Disease

After one week of acclimatization period, letrozole (1 mg/kg) in suspension form (letrozole suspended in 0.5% carboxymethylcellulose (CMC)) was given for 7 weeks to rats for the induction of PCOS. This dose was chosen according to protocols suggested in a published article by Rezvanfar et al. [23]. During this period, the estrous cycle was assessed daily by vaginal cytology (relative proportion of cornified cells, leukocytes, and epithelial cells under light microscopy) and weight variations were recorded weekly. The leaving criteria of the study were based on the cyclicity of estrous cycle, % reduction in weight gain, and the restoration of biochemical parameters.

### 2.8. Experimental Design and Collection of Samples

Animals were divided randomly in 5 groups (*n* = 5). Groups were labeled as I, II, III, IV, and V (Table 1). All the doses were administered daily through oral gavage for seven weeks and vaginal smear was examined daily via colposcopy [24]. After seven weeks of dosing, all the animals were weighed and euthanized after 24 hours of the last dose. The blood sample was collected by cardiac puncture. The serum of each sample was separated out by centrifugation and freezed at −20°C for biochemical analysis and hormonal evaluation. Ovaries were removed and dissected to perform their histopathology. The liver of the rats was preserved for evaluation of antioxidant status [7, 25].

### 2.9. Estrous Cycle Monitoring and Vaginal Smear Cytology

Estrous cycle consists of four stages, e.g., proestrus, estrus, metestrus, and diestrus. To determine these stages of the estrous cycle, the already-reported method was adopted. The proestrus stage consists of nucleated epithelial cells predominated with a small number of leukocytes. The estrus stage of the estrous cycle comprises cornified epithelial cells. Metestrus stages embraced with leukocytes, cornified epithelial cells, and nucleated cornified cells while the diestrus stage consists of mostly leukocytes with more mucus. To assess the different stages of estrous cycle, a small piece of cotton swab was moistened with 0.9% normal saline and samples from the rat vagina were taken. Slides were prepared and stained with methylene blue dye. Vaginal cytology assessment was performed by using a light microscope [26].

### 2.10. Ovarian Histopathological Examination

Rat ovaries of all groups were dissected out and fixed in 10% buffer formalin solution. After fixation, paraffin blocks were made and 5 *μ* thick slices were cut using microtome. Slides were prepared and stained with eosin and hematoxylin stains.

### 2.11. Serum Hormone Analysis

Serum hormone analysis was performed by radioimmunoassay (RIA) and enzyme-linked immunosorbent assay (ELISA) kit methods. The blood sample was taken by cardiac puncture and then, the serum was separated out by cold centrifugation. Serum follicle-stimulating hormone (FSH; ml*μ*/mL), progesterone (ng/dL), prolactin (ng/mL), and testosterone (ng/dL) levels were measured by RIA utilizing the kit of Beckman Coulter, Inc. USA. The serum luteinizing hormone (LH; ml*μ*/mL) concentration was determined by the ELISA method utilizing the kit of Pointe Scientific Inc. USA. Serum estrogen (pg/mL) levels were evaluated by using the kit of ALPCO, USA, and serum insulin (*μ*lU/ml) levels were determined by using the kit of Calbiotech Inc., USA.

### 2.12. Lipid Profile and Liver Function Tests

Various markers of lipid profile, i.e., total serum cholesterol, triglycerides, HDL, and LDL levels were determined. Similarly, different markers of liver function test, i.e., levels of ALT, AST, and ALP along with total and direct bilirubin levels were also evaluated. All these tests were conducted using an automated chemistry analyzer (Microlab-300).

### 2.13. Antioxidant Activity Assays

#### 2.13.1. Determination of Enzyme Activities using Liver Homogenates

It is commonly observed that in polycystic ovarian syndrome, the levels of antioxidant enzymes, e.g., catalase (CAT), reduced glutathione (GSH), and superoxide dismutase (SOD) are altered and therefore were evaluated in the current study. The liver tissues were homogenized to prepare liver homogenates by using 0.1 M phosphate buffer saline (pH 7.4). Supernatant was obtained by centrifuging the mixture at 800 rpm at 4°C for 30 min. This supernatant solution was used for the determination of different antioxidant parameters (CAT, SOD, and GSH) [27].

#### 2.13.2. DPPH Radical Scavenging Assay

Stock solution of DPPH (2.4 mg/100 mL methanol) was prepared. *F*. *indica* extract stock solution (5 mg/mL in methanol) was also made. Different sample dilutions (20, 40, 60, 80, and 100 ppm) were made using the *F*. *indica* extract stock solution. Similarly, 200 mg/L stock solution of BHT (butylated hydroxytoluene) was prepared and using this BHT stock solution, different sample dilutions (20, 40, 60, 80, and 100 ppm) were prepared. Subsequently, added 4 mL of DPPH solution in both plant samples and BHT samples. The mixture was kept at room temperature for 30 min in dark, and the absorbance was recorded at 515 nm using a spectrophotometer. The absorbance of the blank (DPPH radicle without antioxidant) was also recorded [28].

### 2.14. Statistical Analysis

The data were demonstrated as mean ± SEM, and the results of different groups were compared by analysis of variance (ANOVA) using GraphPad Prism 7.04 followed by the Tukey's multiple comparisons test. The results were considered statistically significant when the *p* value was less than 0.05.

## 3. Results

### 3.1. Plant Characterization

#### 3.1.1. Analysis of Phytoconstituents of *F*. *indica* using HPLC

**Figure 1:**
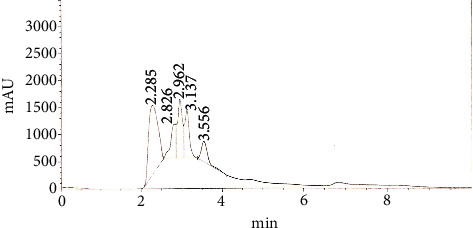
HPLC analysis of ethanolic extract of *Fagonia indica*.

HPLC analysis of ethanolic extract of *F*. *indica* showed the presence of phenolic contents, i.e., synaptic acid (1.603 mg/gm), benzoic acid (2.785 mg/gm), and traces of chlorogenic acid. While, flavonoid contents in ethanolic extract of *F*. *indica* included myricetin (0.672 mg/gm) and quercetin (0.904 mg/gm) (Figure 1)

#### 3.1.2. Total Phenolic Content

The TPC of the ethanolic extract of *F*. *indica* was determined as 109.56 ± 0.124. The TPC is expressed as gallic acid equivalent (GAE).

#### 3.1.3. Total Flavonoid Content

The TFC of the ethanolic extract of *F*. *indica* was determined as 35.10 ± 0.08. The TFC is expressed as catechin equivalent (CE).

### 3.2. Estrous Cycle Monitoring and Vaginal Smear Cytology

**Figure 2:**
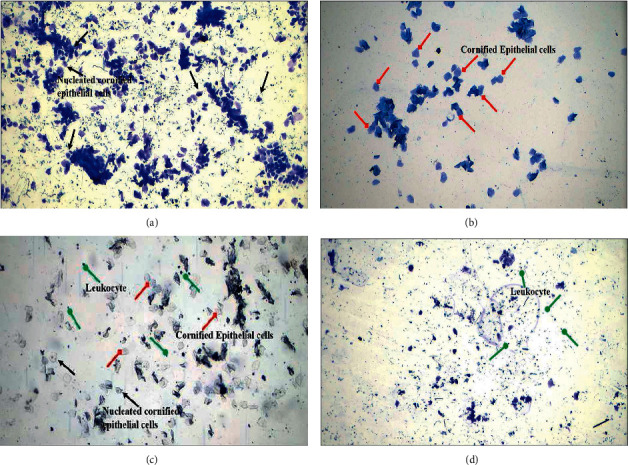
Vaginal smear of healthy rat (normal control group) showing different stages of the estrous cycle. (a) Proestrous phase containing dominantly nucleated cornified epithelial cells (black arrow). (b) Estrous phase characterized by cornified epithelial cells (red arrow). (c) Metestrous phase: in this phase, leukocytes (green arrow), cornified epithelial cells, and nucleated cornified cells are present. (d) Diestrous phase comprises of mostly leukocytes (green arrow).

Throughout the study period, changes of the estrous cycle (proestrus, estrous, metestrus, and diestrus phases) were regular at regular time periods in group I, indicating a normal estrous cycle.  Figure 2 shows the four stages of estrous cycle of a normal healthy rat.

**Figure 3:**
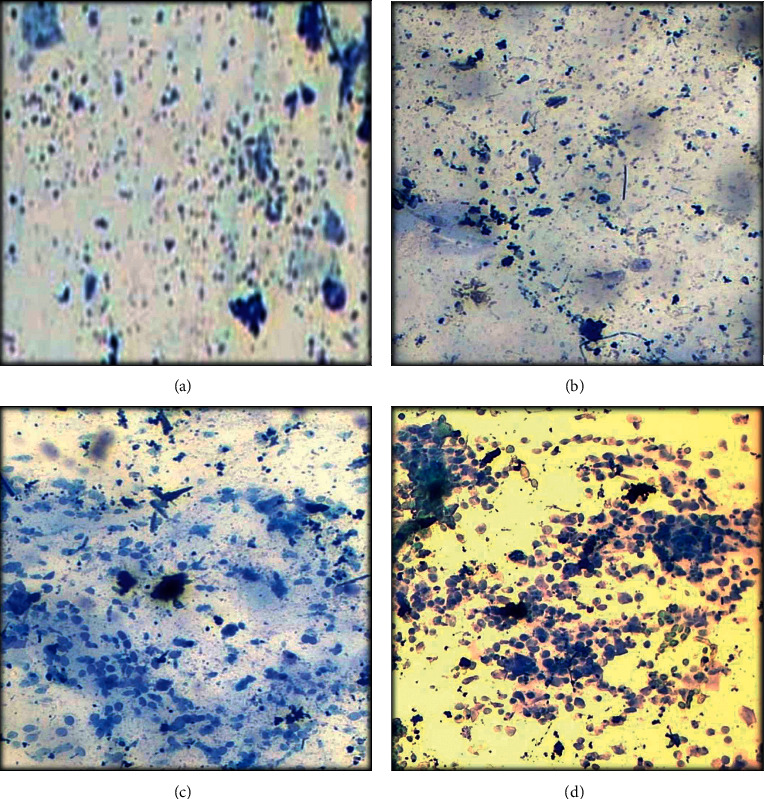
Vaginal smear at different stages of estrous cycle after PCOS induction and treatment. (a) PCOS group with diestrous phase. (b) Metformin treated group with diestrous phase. (c) *Fagonia* extract treated group with estrous phase. (d) Metformin plus *Fagonia*-treated group with an estrous phase of the cycle.

Animals with PCOS delayed the estrous cycle and remained in the diestrus phase for a prolonged time. Animals of group III who were treated with metformin remained in the diestrus phase as most of the cells found in vaginal smear were leukocytes, a characteristic feature of the diestrus phase. Examination of groups IV and V showed the estrous phase of cycle that consists majorly of cornified epithelial cells (Figure 3).

### 3.3. Effect of *Fagonia indica* on Histopathology of Rat Ovaries

**Figure 4:**
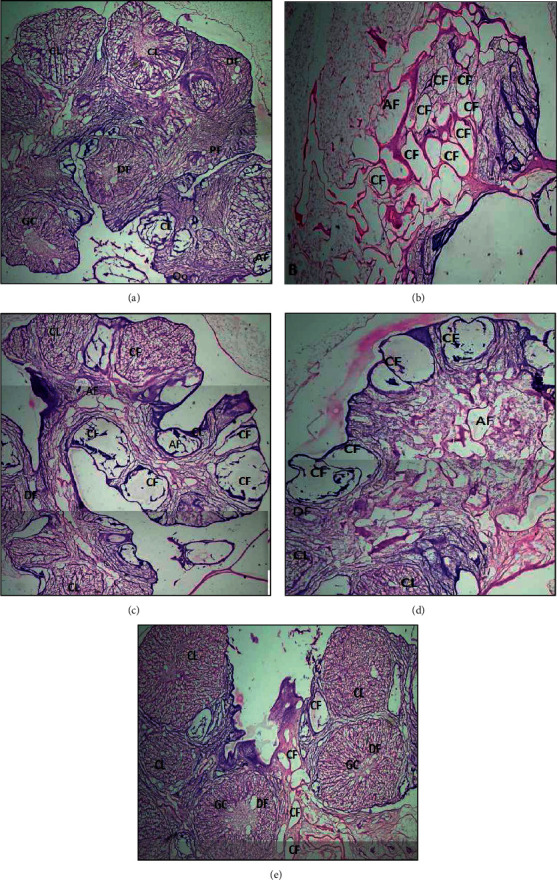
Ovarian cross section stained by hematoxylin-eosin. (a) Section from the control group showing normal morphology with different stages of ovarian follicles. (b) Ovary from PCOS rat containing various cystic follicles. (c) Ovarian section of PCOS rat treated with metformin. (d) Ovary of PCOS rat treated with *Fagonia* extract. (e) Ovary of PCOS rat treated with metformin plus *Fagonia*. CL: corpus luteum; CF: cystic follicles; AF: atretic follicles; DF: developing follicles; PF: primary follicles; GC: granulosa cells; Oo: oocyte. Scale bar is equal to 100 *μ*M. Magnification: 4X.

Histopathological slides of group I ovary exhibited normal architecture with small- to medium-sized anteral follicles, multiple corpus luteum, granulosa cells, oocyte, and various stages of developing follicles (Figure 4(a)). Ovary slides of group II rats presented with cystic follicles and disorganized granulosa cell compartment with irregular thickness of granulosa cells that is the characteristic of atretic anteral follicles. Letrozole-treated ovaries lacked corpus luteum (Figure 4(b)). These changes may be explained by decreased FSH and increased testosterone level in the letrozole-treated group. However, reduction in number of cystic follicles was found metformin and *Fagonia indica*-treated groups, respectively (Figures 4(c) and 4(d)). While, appreciably restored normal anatomy of ovaries were found in the animals of group V (metformin + *Fagonia*) (Figure 4(e)).

### 3.4. Body Weight Changes

**Figure 5:**
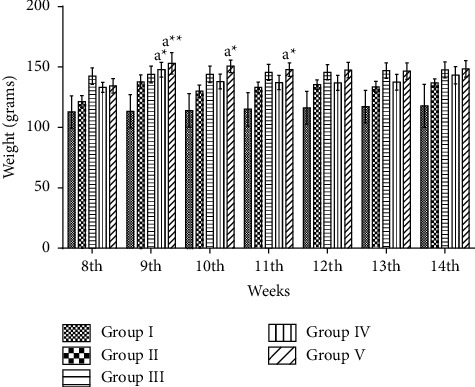
Effect of ethanolic extract of *Fagonia indica* on weekly body weight (grams) in letrozole-induced PCOS rat model. Values are expressed as mean ± SEM (*n* = 5). Comparison among groups were performed by using two-way ANOVA followed by Tukey's multiple comparisons test. ns *p* > 0.05; ^*∗*^*p* < 0.05; ^*∗∗*^*p* < 0.01; ^*∗∗∗*^*p* < 0.001.

At the end of the study period, group II rats generally gained more weight than group I rats. Body weight of control rats were increased by 18% of its original weight, while the weight of PCOS rats was increased by 35%, i.e., almost the double of weight gain of the rats in the control group. The weight difference among all groups was mostly statistically nonsignificant (Figure 5).

### 3.5. Effect of *F*. *indica* on Hormonal Status

**Figure 6:**
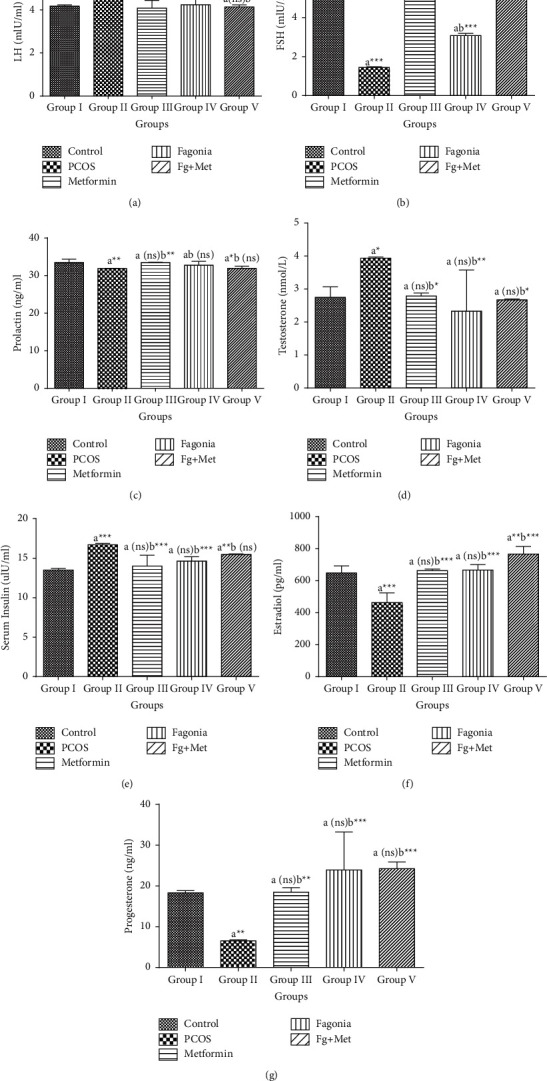
Effects of ethanolic extract of *F*. *indica* on (a) LH, (b) FSH, (c) prolactin, (d) testosterone, (e) insulin, (f) estradiol, and (g) progesterone level in letrozole-induced PCOS rats. Values are expressed as mean ± SEM (*n* = 5). ns: nonsignificant. ^a^Significantly different from Group I (control); ^b^Significantly different from Group II (PCOS). Comparison among groups was made by one-way ANOVA followed by Tukey's multiple comparisons test. ns *p* > 0.05, ^*∗*^*p* < 0.05, ^*∗∗*^*p* < 0.01, ^*∗∗∗*^*p* < 0.001.

Group II (PCOS) showed significant increase (*p* < 0.05) in LH levels as compared to group I (normal control). LH levels were found decreased significantly in group III (*p* < 0.05) and group V, (*p* > 0.05) while a nonsignificant decrease was observed in group IV, when compared with group II (Figure 6(a)).

A significant (*p* < 0.0001) reduction was observed in FSH, estradiol, and progesterone concentrations in group II, when compared with group I. The levels were significantly improved (*p* < 0.0001) in all treated groups as compared with group II (Figures 6(b), 6(f), and 6(g)).

A significant (*p* < 0.01) decrease in serum prolactin concentration was observed in group II (PCOS-induced group) as compared to group I (control group). There was a significant increase in the prolactin level in group III (*p* < 0.01); however, nonsignificant elevation was found in other treated groups (Figure 6(c)).

A significant (*p* < 0.05) increase was observed in testosterone concentration of group II (PCOS control rats) when compared with group I (normal control). The data showed significant reduction in testosterone levels in all treated groups as compared with group II (Figure 6(d)).

A significant (*p* < 0.001) increase was observed in serum insulin concentration of group II, when compared with group I. The data showed significant reduction in group III and IV (*P* < 0.001), while nonsignificant reduction was found in group V, when compared with group II (Figure 6(e)).

### 3.6. Effect of Ethanolic Extract of *F*. *indica* on Lipid Profile

In group II, total cholesterol (TC) levels were significantly increased (*p* < 0.0001) as compared to group I. While treatment groups showed significantly lessened levels of total cholesterol as compared with group II.

In group II, significant increase (*p* < 0.0349) was noticed in the triglycerides level compared with group I. However, there was a significant reduction (*p* < 0.0001) in triglycerides (TG) of group III and nonsignificant reduction was noticed in group IV and V in comparison with group II.

There was a significant decrease (*p* < 0.0001) in high-density lipoprotein (HDL) level of group II in comparison with group I. While a nonsignificant increase in the HDL level was observed in group III, IV, and V when compared with group II.

**Figure 7:**
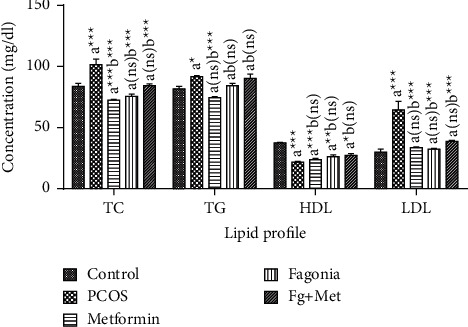
Effect of ethanolic extract of *Fagonia indica* on lipid profile in letrozole-induced PCOS rat model. The data are presented as mean ± SEM (*n* = 5). ns: nonsignificant. ^a^Significantly different from group I (control), ^b^Significantly different from group II (PCOS). Comparisons among groups were made by two-way ANOVA followed by Tukey's multiple comparison test. ns *p* > 0.05; ^*∗*^*p* < 0.05; ^*∗∗*^*p* < 0.01; ^*∗∗∗*^*p* < 0.001.

Low-density lipoprotein (LDL) level in group II was significantly elevated (*p* < 0.0001) as compare to group I. A significant reduction (*p* < 0.0001) was found in the levels of LDL in group III, IV, and V, when compared with group II (Figure 7).

### 3.7. Effects of *F*. *indica* on Liver Functioning Test (LFT)

There was not any statistical difference found in the concentrations of total bilirubin, direct and indirect bilirubin in all groups. Aspartate aminotransferase (AST), alanine transaminase (ALT), and alkaline phosphatase levels were elevated significantly in group II as compared to group I. There was a significant reduction in the levels of AST and ALT in group III, IV, and V when compared with group II. The levels of alkaline phosphatase is found ameliorated in group V while a nonsignificant difference was observed in group III and V as compared to group II (Table 2).

### 3.8. Effects of *F*. *indica* on Antioxidant Enzymes

**Figure 8:**
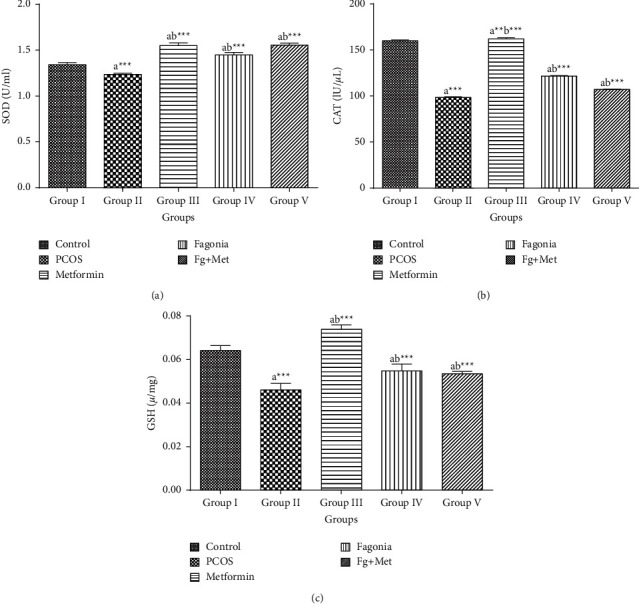
*Fagonia indica* improved the levels of hepatic enzymes (a) SOD, (b) CAT, and (c) GSH. ^a^Significantly different from group I, ^b^Significantly different from group II. The data are presented as mean ± SEM (*n* = 5). Comparison among groups was made by one-way ANOVA followed by Tukey's multiple comparisons test. ns *p* > 0.05; ^*∗*^*p* < 0.05; ^*∗∗*^*p* < 0.01; ^*∗∗∗*^*p* < 0.001.

The levels of antioxidant biomarkers such as superoxide dismutase (SOD), catalase (CAT), and glutathione (GSH) were estimated in liver samples. The levels of all these parameters were decreased (*p* < 0.001) in group II as compared to group I. Results showed that in all the treated groups, oxidative stress biomarkers were increased significantly (*p* < 0.05) when compared with group II (Figures 8(a)–8(c)).

### 3.9. DPPH Assay

Percent inhibition of DPPH by *Fagonia* extract is mentioned in Table 3. The sample is taken in parts per million (ppm). Butylated hydroxytoluene (BHT) was used as a standard for comparison of results.

## 4. Discussion

The ovaries are highly organized female reproductive organs composed of germ cells (eggs or oocytes) and somatic cells (stromal cells, theca cells, and granulosa cells). Interaction of these cells decides the development and formation of oocyte-containing follicles, ovulation of egg, and formation of corpus luteum. Follicular development is regulated by hypothalamic-pituitary-ovarian axis (HPO). In this axis, hypothalamic gonadotropin-releasing hormone (GnRH) stimulates the secretion of pituitary hormones, e.g., FSH and LH [29]. Follicular development is regulated by hypothalamic-pituitary-ovarian axis (HPO). In this axis, hypothalamic gonadotropin-releasing hormone (GnRH) stimulates the secretion of pituitary hormones, e.g., FSH and LH. Hypothalamus stimulates LH synthesis over FSH, results in elevated LH/FSH ratio in PCOS women. Increased level of LH causes the increased production of testosterone that consequently leads to follicular arrest and increased AMH levels [30]. Polycystic ovarian syndrome is very complex endocrine disorder that is most frequently encountered gynecological endocrinopathy among reproductive aged women [31]. Many genetic and environmental factors are responsible for the etiology of this syndrome. Unhealthy lifestyle and diet or any infectious mediators elevate the probability of PCOS. In the case of PCOS, LH pulse frequency is significantly increased, while the FSH production is reduced causing positive feedback on the GnRH pulse frequency. It worsen the condition by releasing more LH [32]. In the case of treatment group, the LH/FSH is decreased and has negative feedback on the GnRH pulse frequency which ultimately decreased the LH levels and increased the production of FSH. One of the factors is insulin resistance that disturbs ovarian function and raises androgen levels leading to anovulation. Gonadotropin-releasing hormone (GnRH), FSH, LH, and prolactin levels are also disturbed in PCOS [33]. Cytochrome P450c17 (CYP17A1) is the key enzyme (rate-limiting enzyme) that limits the sex steroidal production in theca cells of ovary and adrenal cortex of adrenal gland. Strong evidences suggested the ovary as the main source of androgens in PCOS women although in minority of cases, the adrenal gland might also contributed for androgen excess. Neuroendocrine dysregulation may also participate in hyperandrogenism in ovaries that contribute to the pathogenesis of PCOS [32]. There is growing literature illustrating the effect of oxidative stress in pathophysiology of PCOS. Oxidative stress markers are elevated in the body of PCOS women [23]. Oxidative stress is defined as the imbalance between the overproduction of oxidants and the limited reserves of antioxidant defenses. PCOS is also considered as an oxidative state. Oxidative stress is partly associated with the various characteristics of this disease including IR, obesity, hyperandrogenism, and abdominal adiposity. These conditions lead towards the development of oxidative stress [34]. Evidences suggested that many herbal remedies were effective in reducing the oxidative stress, e.g., *Panax ginseng*, *Olea europaea*, and many other plants that have the antioxidant potential [35, 36]. A vitamin-like nutrient called coenzyme Q10 (CoQ10) also has a remarkable antioxidant potential. It has a positive effect on the reproductive health. CoQ10 is also involved in the expression of genes such as proliferation cell nuclear antigen (PCNA) and follicle‐stimulating hormone receptor (FSHR) that are responsible for the folliculogenesis [37].

Herbal drugs have shown excellent results in management of PCOS having steady therapeutic effects with least side effects. They increase immunity of body and regularize menstrual cycle [16]. The ethanolic extract of *Fagonia indica* contains different phenolic and flavonoid phytoconstituents that have potential to treat signs and symptoms of PCOS.

The PCOS rat model was successfully developed by administration of letrozole. It is a nonsteroidal and highly potent aromatase inhibitor [38]. Current study showed an increase in weight gain of PCOS rats as compared to normal control rats and clear difference between normal control rat ovaries and PCOS rat ovaries was observed in histopathological evaluation. Furthermore, vaginal smear cytology expressed that letrozole induced the cysts in ovaries of rats as PCOS rats remained in diestrus phase for a prolonged time. Letrozole also reduced the levels of catalase (CAT), superoxide dismutase (SOD), and glutathione (GSH) in the body [25]. Previously, increased oxidative status has been determined in the systemic circulation and follicular fluids of PCOS rats [39]. Imbalanced reproductive hormones and insulin resistance is the key characteristics in PCOS patients and were responsible for the imbalance in energy homeostasis, development of adiposity, and weight gain [40].

Previously, *F*. *indica* has shown antioxidant, antiobesity, antilipoprotein and antidiabetic activities. It regularized the imbalanced reproductive hormones as well. So, the present study was aimed to explore its therapeutic potential on PCOS rat model. *F*. *indica* have antioxidant potential due to the presence of its flavonoid phytoconstituents [41]. Quercetin and myricetin are two flavonoids that were identified in the ethanolic extract of *Fagonia indica* and are known potent antioxidants [25, 42]. Antioxidant activity is due to the presence of OH group on the phenyl ring of quercetin and myricetin and is dependent upon the presence of OH groups [43]. Quercetin is known to arrest the oxidative stress by triggering the nuclear factor (erythroid-derived 2)-like 2/antioxidant response element (Nrf2-ARE) pathway and stimulating the expression of phase II antioxidant enzymes such as glutathione s-transferase (GST), CAT, NAD(P)H: quinone oxidoreductase 1 (NQO1), SOD, and glutathione peroxidase (GPx) [44]. The results of current study are in-line with the abovementioned inferences and showed that *F*. *indica* elevated the levels of CAT, GSH, and SOD enzymes.

Histology of vaginal smear is the key determinant of ovarian physiology. Vaginal smear test is indicative of disease induction in group II as the leukocytes were present abundantly showing the diestrus stage of estrous cycle. PCOS rats remained for a prolonged period in their diestrus stage. While, treatment with *F*. *indica* abolished the irregularity of estrous cycle and controlled the cyclicity. These changes might be attributed to decreased FSH leveled and increased testosterone leveled in PCOS group. Treatment with *F*. *indica* enhanced the levels of FSH and reduced the levels of testosterone. Furthermore, combination treatment exhibited remarkable outcomes in group V by restoring the nearly normal physiology of ovaries. Previous evidences suggested that quercetin possessed innate ability to rectify hormonal imbalance and subsequent metabolic disorder that occurred in PCOS [31].

Serum LH levels are increased in PCOS because of disrupted hypothalamic-pituitary axis that activates PI3K/Akt pathway causing the overexpression of ovarian CYP17A1 gene along with 17-*α* hydroxylase enzyme levels, which is used in the reaction to synthesize the androgen from progesterone [45]. Previous research data have shown the ability of quercetin to inhibit the pathway of androgen biosynthesis due to the LH. Lowering effects of quercetin on the levels of LH and testosterone are associated to resistin that is important for steroidogenesis [31]. Current study showed that treatment with *F*. *indica* nearly normalized the LH levels.

Current study showed that *F*. *indica* reduced serum levels of total cholesterol, TGs, and LDL while, caused an increase in HDL levels. This may be attributed to quercetin content of the ethanolic extract of *Fagonia indica* [31]. Proposed mechanism for quercetin to lower the triglyceride level in obese patient who consumes diet overloaded with free fatty acids (FFAs) (highly saturated food) has been reported by Kim et al. [46]. Quercetin is known to restrict the hepatic triglycerides accumulation by enhancing the hepatic mitochondrial oxidative metabolic capacity which subsequently causes the reduction of FFA-induced lipid peroxidation. It also prevents mitochondrial damage and hepatic lipid accumulation [46]. PCOS patients are hyperglycemic because of insulin resistance. Evidence suggested that *F*. *indica* also have hypoglycemic effect [47]. Current study showed that treatment with *F*. *indica* reduced insulin resistance.

Furthermore, this study can be extended to find out the effects of ethanolic extract of *Fagonia indica* at the molecular and genetic levels. Future studies may also emphasize on identification of phytochemical constituents responsible for the found effect.

## 5. Conclusion

Current study showed that the ethanolic extract of *Fagonia indica* had the potential to ameliorate polycystic ovarian syndrome at the dose of 500 mg/kg which is statistically comparable with metformin. This property might be attributed to antioxidant activity and improvement in liver enzymes and lipid profile, along with amelioration of hormonal enzymes by *F*. *indica*.

## Figures and Tables

**Table 1:** Experimental design.

Groups	Treatment
Group I (vehicle control)	Aqueous solution of carboxymethyl cellulose (CMC 0.5%)
Group II (PCOS/disease control)	Letrozole (1 mg/kg b.w.) dissolved in 0.5% carboxymethyl cellulose
Group III (metformin/standard)	Metformin (20 mg/kg b.w.) to letrozole-induced PCOS rats
Group IV (*Fagonia* extract)	*F*. *indica* (500 mg/kg b.w.) to letrozole-induced PCOS rats
Group V (*Fagonia* + metformin)	*F*. *indica* (500 mg/kg b.w.) + metformin (20 mg/kg b.w.) to letrozole-induced PCOS rats

**Table 2:** Effect of *F*. *indica* on bilirubin (total, direct, and indirect), ALT, AST, and alkaline phosphatase levels. ns: nonsignificant. ^a^Significantly different from group I; ^b^Significantly different from group II. The data are presented as mean ± SEM (*n* = 5). Comparisons among groups were made by two-way ANOVA followed by Tukey's multiple comparison test. ns *p* > 0.05; ^*∗*^*p* < 0.05; ^*∗∗*^*p* < 0.01; ^*∗∗∗*^*p* < 0.001.

Parameters	Group I	Group II	Group III	Group IV	Group V
Total bilirubin (mg/dl)	2.70 ± 0.07	2.73 ± 0.01	2.73 ± 0.01	2.73 ± 0.01	2.70 ± 0.07
Direct bilirubin (mg/dl)	2.57 ± 0.01	2.87 ± 0.01	2.57 ± 0.01	2.87 ± 0.01	2.57 ± 0.01
Indirect bilirubin (mg/dl)	0.70 ± 0.002	0.69 ± 0.002	0.69 ± 0.002	0.70 ± 0.002	0.69 ± 0.002
(ALT) (U/l)	22.26 ± 0.02	28.80 ± 0.01^a^^*∗∗∗*^	25.78 ± 0.2^ab^^*∗∗∗*^	18.47 ± 0.02^ab^^*∗∗∗*^	27.69 ± 0.02^ab^^*∗∗∗*^
(AST) (U/l)	179.62 ± 0.09	189.62 ± 0.09^a^^*∗∗∗*^	175.20 ± 0.88^ab^^*∗∗∗*^	178.50 ± 0.01^ab^^*∗∗∗*^	174.90 ± 0.01^ab^^*∗∗∗*^
Alkaline phosphatase (U/l)	20.45 ± 0.02	24.15 ± 0.11^a^^*∗∗∗*^	23.88 ± 0.06^a^^*∗∗∗*^^b^ (ns)	18.60 ± 0.01^ab^^*∗∗∗*^	23.81 ± 0.01^a^^*∗∗∗*^^b^ (ns)

**Table 3:** DPPH scavenging activity of ethanolic extract of *F*. *indica*.

BHT standard		Ethanolic extract of *Fagonia indica*	
Sample ppm	Scavenging %	Sample ppm	Scavenging %
20	55.27	20	46.83
40	63.5	40	49.75
60	76.31	60	54.23
80	84.64	80	57.56
100	88.6	100	59.33

BHT: butylated hydroxytoluene; DPPH: 2,2-diphenylpicrylhydrazyl.

## Data Availability

The data used to support the findings of this study are available from the corresponding author upon reasonable request.
